# Activation of kinin B1 receptor evokes hyperthermia through a vagal sensory mechanism in the rat

**DOI:** 10.1186/1742-2094-9-214

**Published:** 2012-09-13

**Authors:** Sébastien Talbot, Helaine De Brito Gariépy, Julien Saint-Denis, Réjean Couture

**Affiliations:** 1 Department of Physiology, Faculty of Medicine, Université de Montréal, C.P. 6128, Succursale Downtown, Montréal, QC H3C 3J7, Canada

**Keywords:** Kinin B1 receptor, Diabetes, Hyperthermia, Inflammation, Bradykinin, NO, Sensory C-fibers, Thermoregulation

## Abstract

**Background:**

Kinins are mediators of pain and inflammation. Their role in thermoregulation is, however, unknown despite the fact the B1 receptor (B1R) was found implicated in lipopolysaccharide (LPS)-induced fever. The aim of this study was to investigate the mechanism by which peripheral B1R affects body core temperature in a rat model known to show up-regulated levels of B1R.

**Methods:**

Male Sprague–Dawley rats received streptozotocin (STZ, 65 mg/kg; i.p.) to enhance B1R expression. Control rats received the vehicle only. One week later, rectal temperature was measured in awake rats after i.p. injection of increasing doses (0.01 to 5 mg/kg) of des-Arg^9^-Bradykinin (BK) and Sar-[D-Phe^8^]des-Arg^9^-BK (B1R agonists) or BK (B2R agonist). The mechanism of B1R-induced hyperthermia was addressed using specific inhibitors and in rats subjected to subdiaphragmatic vagal nerve ligation. B1R mRNA level was measured by quantitative Real Time-polymerase chain reaction (qRT-PCR) and B1R was localized by confocal microscopy.

**Results:**

B1R agonists (0.1 to 5 mg/kg) showed transient (5- to 30-minute) and dose-dependent increases of rectal temperature (+1.5°C) in STZ-treated rats, but not in control rats. BK caused no effect in STZ and control rats. In STZ-treated rats, B1R agonist-induced hyperthermia was blocked by antagonists/inhibitors of B1R (SSR240612), cyclooxygenase-2 (COX-2) (niflumic acid) and nitric oxide synthase (NOS) (L-NAME), and after vagal nerve ligation. In contrast, COX-1 inhibition (indomethacin) had no effect on B1R agonist-induced hyperthermia. In STZ-treated rats, B1R mRNA was significantly increased in the hypothalamus and the vagus nerve where it was co-localized with calcitonin-gene-related peptide in sensory C-fibers.

**Conclusion:**

B1R, which is induced in inflammatory diseases, could contribute to hyperthermia through a vagal sensory mechanism involving prostaglandins (via COX-2) and nitric oxide.

## Background

Heat- or hyperthermia-generating processes are generally ascribed to peripherally formed cytokines which convey information to the hypothalamic preoptic area via the *organum vasculosum laminae terminalis*[1]. This pathway is associated with high circulating levels of cytokines from the immune system [2]. Hyperthermia can also be induced even with low circulating levels of cytokines through a neuronal mechanism involving direct activation of the vagus nerve [3]. Indeed, vagal sensory afferents represent an important communication pathway between the immune system, the inflammatory site and the brain [4]. Subdiaphragmatic vagotomy was shown to abolish fever [5] and central induction of interleukin-1β (IL-1β) mRNA [6] after intraperitoneal (i.p.) injection of IL-1β or lipopolysaccharide (LPS).

Kinins are vasoactive peptides involved in pain and inflammation [7-13]. They act through the activation of two G-protein-coupled receptors denoted as B1 (B1R) and B2 (B2R) [14,15]. The B2R is widely and constitutively expressed in central and peripheral tissues and is activated by bradykinin (BK) and Lys-BK. Their metabolites, des-Arg^9^-BK and Lys-des-Arg^9^-BK, are the preferential agonists of B1R [15,16]. The latter is virtually absent in healthy condition but up-regulated after exposure to pro-inflammatory cytokines, LPS and oxidative stress [9,16-19]. LPS, an endotoxin derived from the cell wall of Gram-negative bacteria, induces fever through cytokine release and Toll-like receptor 4 activation in several species, notably in rats, mice and rabbits [1,20,21]. When injected intracerebroventricularly (i.c.v.), B2R antagonist curtailed the early phase (0 to 2 h) of the febrile response induced by LPS while B1R antagonist inhibited the late phase (4 to 6 h) [22]. These authors also demonstrated that a 24-h pre-treatment with LPS reduced the febrile response induced by BK but enhanced that induced by the B1R agonist des-Arg^9^-BK injected i.c.v. In rabbits, BK (i.c.v.) also increased rectal temperature dose-dependently, which was partly mediated by prostaglandins (PGs) [23]. Similarly, the stimulation of rat brain B2R caused hyperthermia [24], an effect absent in animals with a bilateral lesion of the hypothalamic paraventricular nucleus [25]. The role of peripheral kinin receptors in fever, however, remains unknown.

Kinin B1R is involved in the main cardinal signs of inflammation, such as pain [8,11], edema and increased vascular permeability [13,17,26] and vasodilatation [27,28] through the release of pro-inflammatory cytokines (IL-1β, IL-6) and other mediators (NO, substance P and PGs) [8,11,12]. Surprisingly, the role of kinin B1R in the regulation of body core temperature has never been investigated in the periphery. As B1R is virtually absent in control rats, we used streptozotocin (STZ)-treated rats as a model to induce B1R expression [10,29-31]. This study was then undertaken to determine whether intraperitoneal activation of B1R with selective agonists enhances rectal temperature through a vagal afferent pathway. Pharmacological treatments with inhibitors were administered to determine the contribution of inflammatory mediators (NO, PGs). The expression of B1R in the hypothalamus and vagus nerve was also determined by quantitative real-time PCR (qRT-PCR) and confocal microscopy.

## Methods

### Experimental animal and care

All research procedures and the care of the animals were in compliance with the guiding principles for animal experimentation as enunciated by the Canadian Council on Animal Care and were approved by the Animal Care Committee of our University. Male Sprague–Dawley rats (200 to 225 g; Charles River, St-Constant, QC, Canada) were housed two per cage under controlled conditions of temperature (23°C) and humidity (50%), on a 12 h light–dark cycle and allowed free access to a normal chow diet (Charles River Rodent) and tap water.

### STZ treatment

STZ is a chemotherapeutic agent of the glucosamine-nitrosourea class, commonly used to treat human Langerhans islet cancer. Since STZ is structurally similar to glucose, it is taken up by glucose transporter 2 (GLUT2) in pancreatic β-cells leading to their destruction and, thereby, to insulin deficit and hyperglycemia. This condition mimics human type 1 diabetes.

Rats were injected under low light with freshly prepared STZ (65 mg/kg, i.p.; Zanosar, McKesson, Montreal, QC, Canada) [10]. Age-matched controls were injected with the vehicle (sterile saline 0.9%, pH 7.0). Blood glucose was measured with a commercial blood glucose-monitoring kit (Accusoft; Roche Diagnostics, Laval, QC, Canada) from a drop of blood obtained from the tail vein, in non-fasting animals. The impact of one-week diabetes was assessed on body weight (g), water intake (ml/day/rat) and food consumption (g/day/rat).

A total volume of 250 ml of water and 250 grams of chow diet were made available for daily consumption by two rats per cage; 24 h later the residual amount of water and food was calculated and subtracted from the original amount and divided by two. Thereafter, water bottles were filled up again to 250 ml and food was weighed at 250 grams.

### Measurement of body temperature

Rat body temperature was measured before (0 minute) and after drug injections (5, 10, 15, 30 and 60 minutes) with a lubricated flexible digital thermometer delicately inserted into the rat rectum (2.5 cm) for 10 sec. Experiments were conducted daily at 10:00 A.M. by two experienced investigators. Rats were trained to the procedure in a quiet room during the week preceding experiments. STZ-treated and control rats were pre-treated or not with different drugs described in the following section.

### Experimental protocols in whole animals

Des-Arg^9^-BK (DABK) [14] and the peptidase resistant Sar-[D-Phe^8^des-Arg^9^-BK (SDABK) [32] were used as selective B1R agonists (0.01 to 5 mg/kg) to evaluate the effect of intraperitoneal B1R stimulation on body temperature in control and STZ-treated rats. BK (1 mg/kg) was used as a B2R agonist [14]. The contribution of NO and PGs in B1R-induced hyperthermia was evaluated after 2 h pre-treatment with (a) L-NG-Nitroarginine Methyl Ester (L-NAME) (30 mg/kg, i.p.), a nitric oxide synthase (NOS) inhibitor [33], (b) indomethacin (10 mg/kg, i.p.), a non-steroidal anti-inflammatory inhibitor [34], or (c) niflumic acid (15 mg/kg, i.p.), a selective cyclooxygenase-2 (COX-2) inhibitor [34]. SSR240612 [(2R)-2-[((3R)-3-(1,3-benzodioxol-5-yl)-3-[[(6-methoxy-2-naphthyl)sulfonyl]amino]propanoyl)amino]-3-(4-[[2R,6S)-2,6-dimethylpiperidinyl]methyl]phenyl)-N-isopropyl-N-methylpropanamide-hydrochloride], a highly potent and selective B1R antagonist [35] was administered by gavage (10 mg/kg) 3 h prior to SDABK to ascertain the B1R mediated response on hyperthermia. SSR240612 was obtained from Sanofi-Aventis R&D (Montpellier, France), dissolved in dimethylsulfoxide (DMSO, 0.5% v/v), ethanol (5% v/v) and Tween-80 (5% v/v) and then completed with distilled water [7]. DABK was purchased from Bachem Bioscience, Inc. (King of Prussia, PA, USA) and diluted in sterile saline. SDABK was synthesized at the Biotechnology Research Institute, National Research Council of Canada (Montreal, QC, Canada) and diluted in sterile saline. BK and L-NAME were diluted in sterile saline, while niflumic acid and indomethacin were diluted in 5% DMSO and 95% ethanol, respectively. Unless stated otherwise, other reagents were purchased from Sigma-Aldrich Canada, Ltd (Oakville, ON, Canada).

### Subdiaphragmatic vagal ligation

To investigate the role of the vagus nerve in B1R-induced hyperthermia, rats underwent subdiaphragmatic vagal nerve ligation [36]. Under isoflurane anesthesia and after a midline laparotomy, the stomach and posterior subdiaphragmatic vagal trunks were exposed, and the proximal parts were ligated with 4–0 silk. Sham-operated rats had the same surgery; the vagus nerve was exposed but not ligated. On the day of surgery and for the two subsequent days, rats received the antibiotics trimethoprime and sulphadiazine (Tribrissen 24%, 30 mg/kg, subcutaneously (s.c.), Schering Canada, Inc., Pointe Claire, QC, Canada) and the analgesic ketoprofen (Anafen, 5 mg/kg, s.c., Merial Canada, Inc., Baie d'Urfé, QC, Canada). Rats were housed in individual plastic chambers (40 X 23 X 20 cm) in the same standard conditions; they had free access to water and food and were allowed to recover for one week before STZ administration.

### Confocal microscopy

#### Tissue preparation for microscopy

One-week STZ and vehicle-treated rats were anesthetised with CO_2_ inhalation and decapitated. A portion of the subdiaphragmatic vagus nerve (2.5 cm) was removed, frozen in 2-methylbutane (cooled at −40°C with liquid nitrogen) and stored at −80°C. The vagus nerve was mounted in a gelatin block and serially cut into 20-μm thick coronal sections with a cryostat. Sections were thaw-mounted on 0.2% gelatin-0.033% chromium potassium sulfate-coated slides and kept at −80°C for one month to allow sections to adhere to the coverslip glasses.

#### Slide preparation

On the day of the experiments, sections were thawed at room temperature for 10 minutes to enhance section adhesion. Slides were washed for 10 minutes in phosphate buffered saline (PBS) (pH 7.4), fixed in PBS 4% paraformaldehyde and washed three times (5 minutes). Then, sections were permeabilized for 45 minutes in PBS 0.5% Triton X-100.

#### Immunolabeling protocol

Slides were incubated with a blocking buffer (PBS supplemented with 0.5% Triton X-100, 3% bovine serum albumin (BSA) and 3% donkey serum) to prevent non-specific labeling. Primary antibodies were diluted in the blocking buffer. To generate the B1R antibody, an epitope of 15 amino acids (VFAGRLLKTRVLGTL) localized in the C-terminal part of the B1R protein was injected into rabbits (Biotechnology Research Institute, Montreal, QC, Canada). Care was taken to avoid an epitope sequence region similar to B2R or other rodent proteins. The specificity of this antibody used at 1:1,500 dilution [37] was confirmed by the absence of the 37 kDa band (putative molecular weight of rat B1R) with the pre-immune serum or with immune serum in B1R knock-out mice renal tissues (data not shown). Mouse anti-calcitonin-gene-related peptide (CGRP) (Chemicon, Hornby, ON, Canada) was diluted at 1:2,000 and used as a specific marker of peptidergic C-fibers [31]. Secondary antibodies were alexa fluor 488 anti-rabbit (Chemicon) diluted 1:1,000 and rhodamine anti-mouse (Chemicon) diluted 1:1,000 [31]. Slides were washed three times (5 minutes), mounted with coverslips, fixed with Vectashield (Invitrogen Life technologies, Burlington, ON, Canada) (12 h at room temperature) and stored at −4°C until examination under a confocal microscope (Leica Confocal Microscope, Richmond Hill, ON, Canada).

#### SYBR green-based quantitative RT-PCR

Control and one-week STZ-treated rats were anesthetized with CO_2_ inhalation and decapitated. Subdiaphragmatic vagus nerve (2.5 cm) and hypothalamus (10 mg of tissue) were identified, carefully dissected out and put in RNA*later* stabilization reagent (QIAGEN, Valencia, CA, USA). Protocols for mRNA extraction, cDNA generation, SYBR green-based quantitative RT-PCR and quantification were described elsewhere [10]. The PCR conditions were as follows: 95°C for 15 minutes, followed by amplification cycles at 94°C for 15 s, 60°C for 30 s and 72°C for 30 s. The Vector NTI-designed RT-PCR primer pairs used in this study are presented in Table 1.

**Table 1:** qPCR primer pairs used in this study

	**Sequences**			**Position**			**GenBank**
18S forward	5'	TCA ACT TTC GAT GGT AGT CGC CGT	3'	363	-	386	X01117
18S reverse	5'	TCC TTG GAT GTG GTA GCC GTT TCT	3'	470	-	447	
B1 receptor forward	5'	GCA GCG CTT AAC CAT AGC GGA AAT	3'	367	-	391	NM_030851
B1 receptor reverse	5'	CCA GTT GAA ACG GTT CCC GAT GTT	3'	478	-	454	

#### Statistical analysis

Data were expressed as the means ± S.E.M. of values obtained from *n* rats. Statistical significance was determined with unpaired Student’s *t*-test or with one-way analysis of variance (ANOVA) followed by *post-hoc* Bonferroni test for multiple comparisons. Only probability (p) values less than 0.05 were considered to be statistically significant.

## Results

### Diabetic status and B1R mRNA expression

**Figure 1:**
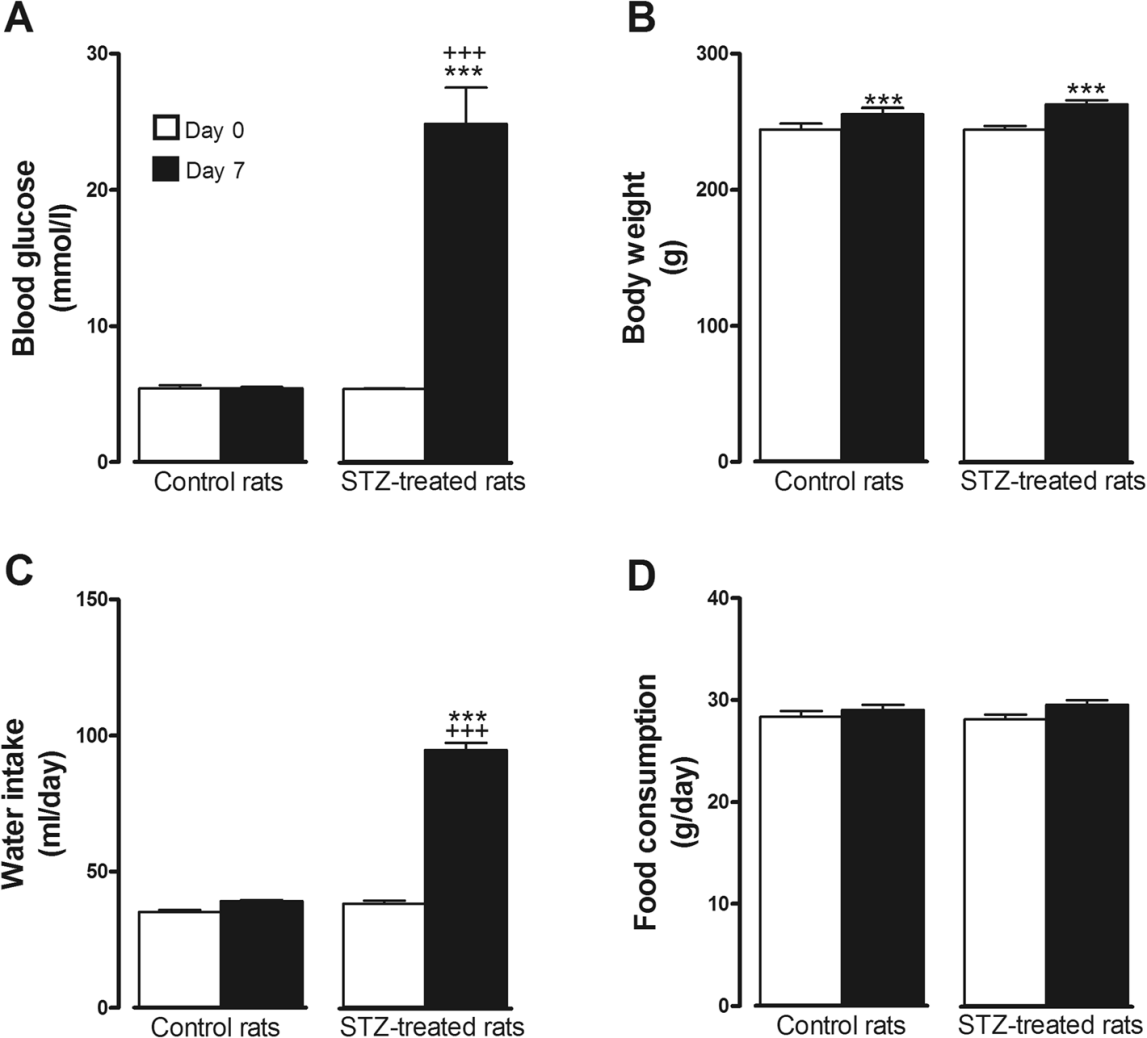
**Physiological parameters in control and STZ-treated rats.** Values of (**A**) blood glucose (mmol/l); (**B**) body weight (g); (**C**) water intake (ml/day); and (**D**) food consumption (g/day) before (Day 0) and after (Day 7) STZ treatment (65 mg/kg, i.p.) or its vehicle (Control). Statistical comparison is indicated between Day 0 and Day 7 (***P <0.001) and between control and STZ-treated rats on Day 7 (+++*P* <0.001). n = 5 to 7 rats.

**Figure 2:**
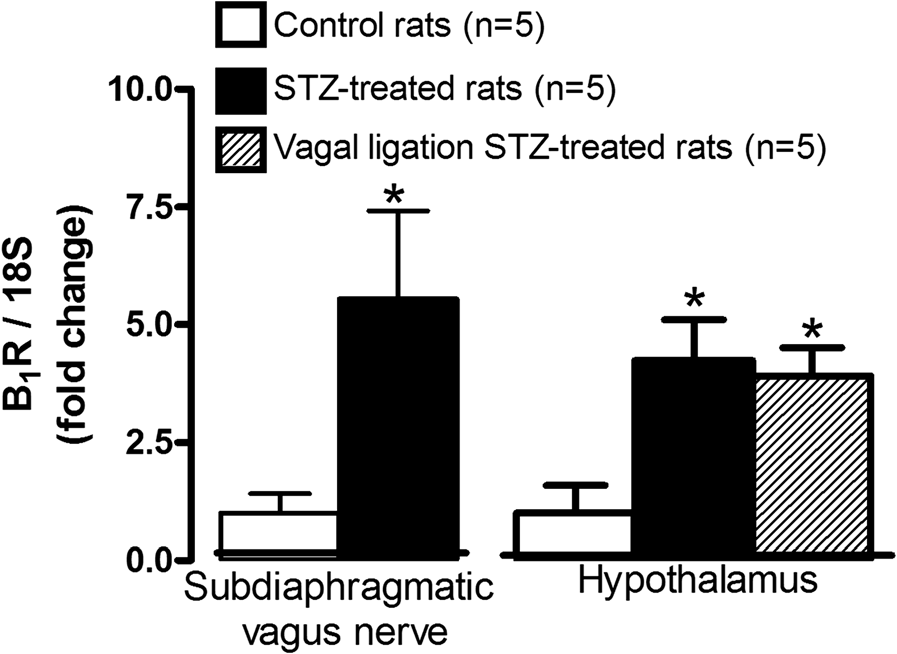
**B1R mRNA levels in the subdiaphragmatic vagus nerve and hypothalamus of control and STZ-treated rats.** The impact of vagal nerve ligation is also shown on hypothalamic B1R mRNA level. Rat 18S was used as a housekeeping gene for quantification. Comparison with control is indicated by * *P* <0.05. n = 5 rats.

Blood glucose, body weight, water intake and food consumption were measured to confirm the diabetic status of STZ-treated rats. As expected, a significant increase in blood glucose and water intake occurred in one-week STZ rats when compared to age-matched control animals. However, body weight gain and food consumption remained unaffected (Figure1). B1R mRNA levels were significantly enhanced (four- to five-fold) in the subdiaphragmatic vagus nerve and hypothalamus of STZ-treated rats when compared to control rats (Figure2). The up-regulation of B1R mRNA was not significantly affected by vagal nerve ligation in STZ-treated rats (Figure2).

### B1R localization in the vagus nerve

**Figure 3:**
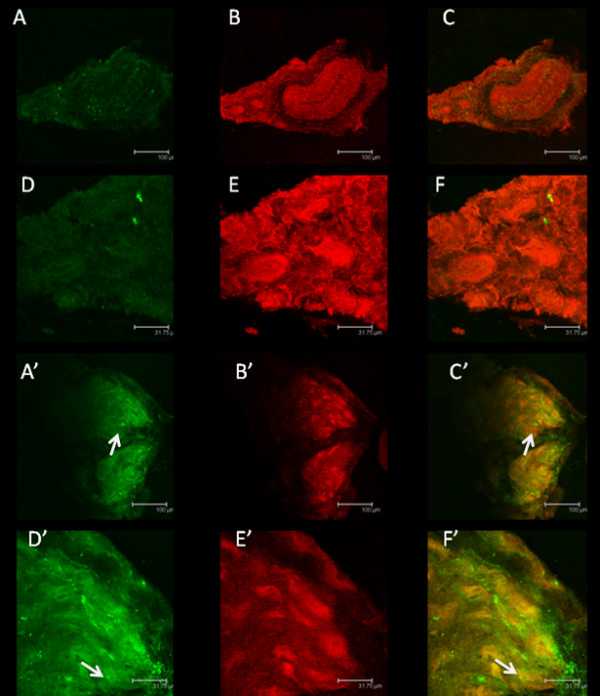
**Immunolocalization of B1R.** Shown are confocal microscopy pictures of coronal sections of subdiaphragmatic vagus nerve isolated from control rats (**A**-**F**) and STZ rats (A’-F’). B1R (**A**-**A**’, **D**-**D**’) was labeled with anti-B1R (green spots, arrows). Peptidergic C-fibers (**B**-**B**’, **E**-**E**’) were labeled with anti-CGRP (red) and overlay pictures (yellow) showing co-localization were shown in **C**-**C**’ and **F**-**F**’. Images are representative of at least four sections from four rats per group. Scale bar = 100 (**A**-**C**, **A**’-**C**’) or 31.8 μm (**D**-**F**, **D**’-**F**’).

**Figure 4:**
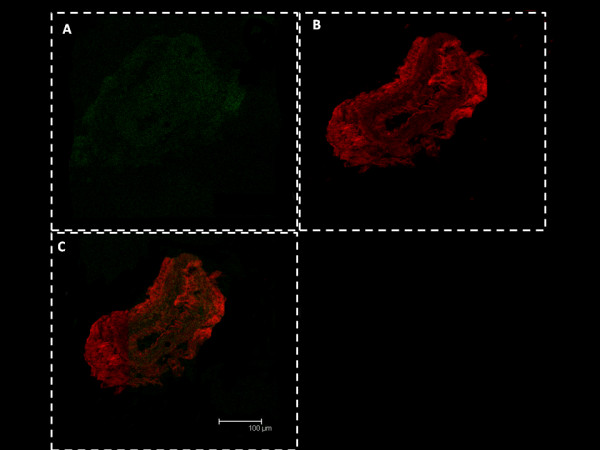
**Specificity of B1R antibody for immunolocalization.** Shown are confocal microscopy pictures of coronal sections of subdiaphragmatic vagus nerve isolated from STZ rats labeled with pre-immune anti-B1R (**A**, green) and anti-CGRP (**B**, red). Picture overlay is presented in panel **C** showing no evidence of co-localization (no yellow color). Images are representative of at least four sections from three rats. Scale bar = 100 μm.

B1R immunostaining was almost undetectable in the control subdiaphragmatic vagus nerve (Figure3A, D), whereas it was markedly enhanced in STZ-treated rat sections (Figure3A', D'). Moreover, B1R was found partly co-localized with CGRP-expressing sensory C-fibers of the vagus nerve in STZ rat (Figure3C', F'). The specificity of B1R labeling was confirmed by the absence of co-localization (no yellow color) with the pre-immune anti-B1R serum (Figure4).

### Effect of B1R stimulation on body temperature in STZ-treated rats

**Figure 5:**
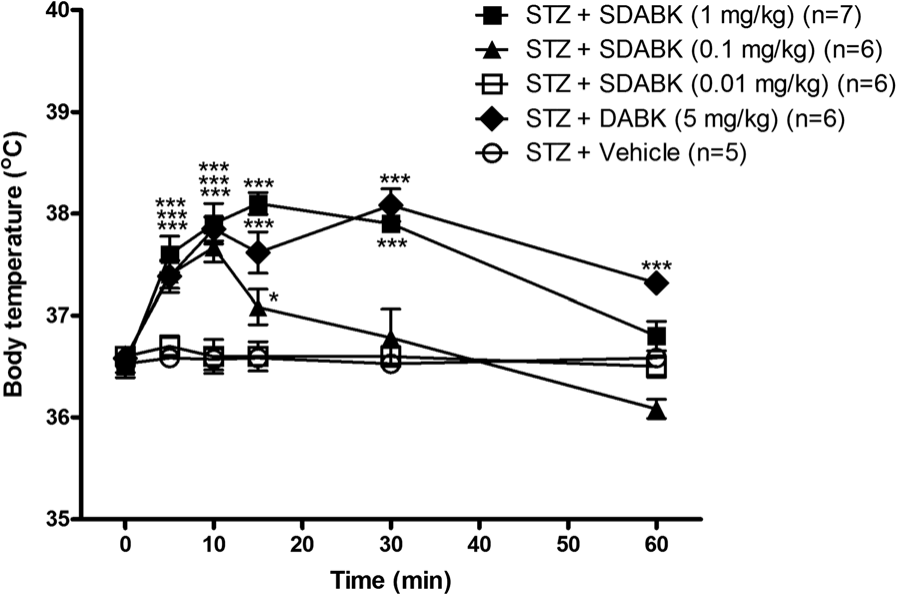
**Rectal temperature changes to B1R agonists.** Two B1R agonists, SDABK (0.01, 0.1, 1 mg/kg) or DABK (5 mg/kg), were injected intraperitoneally in STZ rats. Statistical comparison with STZ + vehicle (*) is indicated by * *P* <0.05 and *** *P* <0.001. n = 5 to 7 rats.

**Figure 6:**
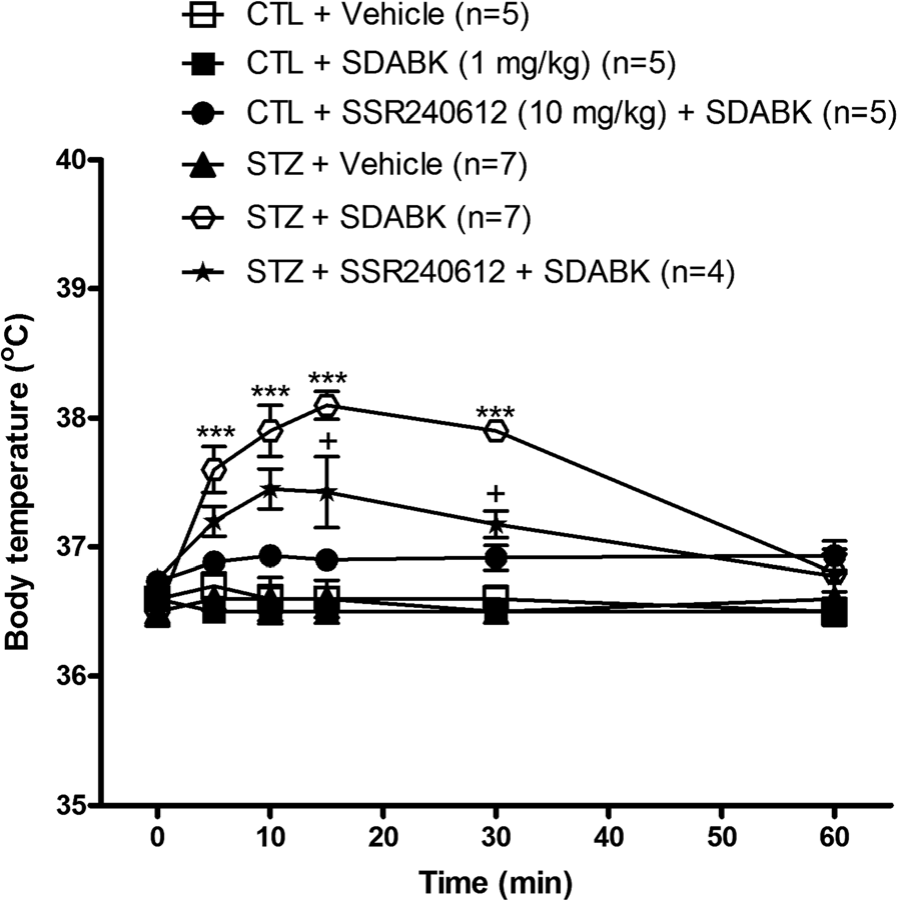
**Inhibitory effect of B1R antagonist on B1R agonist-induced increased rectal temperature.** B1R agonist (SDABK, 1 mg/kg) or its vehicle was injected intraperitoneally in STZ and control rats pre-treated (3 h earlier) with a selective B1R antagonist (SSR240612, 10 mg/kg, per gavage ) or its vehicle. Statistical comparison with STZ + vehicle (*) or with STZ + SDABK (+) is indicated by + *P* <0.05; *** *P* <0.001. n = 4 to 7 rats.

Three doses of the B1R agonist SDABK and one dose of the agonist DABK were injected i.p. in one-week STZ-treated rats to assess their impact on body temperature (Figure5). The dose of 0.01 mg/kg SDABK had no effect on body temperature while the dose of 0.1 mg/kg SDABK increased significantly body temperature at 5, 10 and 15 minutes post-injection. The dose of 1 mg/kg SDABK caused a greater effect (+1.5°C) which peaked at 10 to 15 minutes post-injection and persisted at least 30 minutes in STZ-treated rats. The latter response was similar to that produced by 5 mg/kg DABK, whose response was, however, still significant at 60 minutes. Vehicle had no effect on body temperature in STZ-treated rats (Figure5). Vehicle and 1 mg/kg SDABK had no significant effect on body temperature in control rats (Figure6). Baseline temperature (time 0 minute) was not significantly different between control and STZ-treated rats (Figure6).

**Figure 7:**
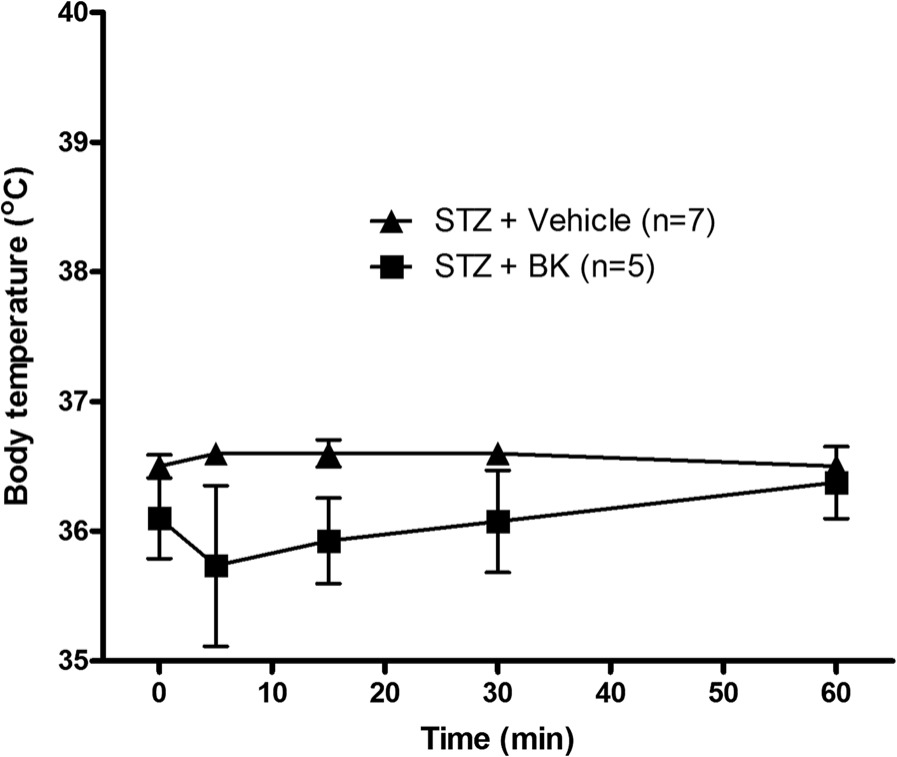
**Rectal temperature changes to B2R agonist.** Bradykinin (1 mg/kg) was injected intraperitoneally in STZ rats. n = 5 to 7 rats. Differences between the two curves were not statistically significant.

The hyperthermic response induced by 1 mg/kg SDABK in STZ-treated rats was significantly reduced by the selective B1R antagonist SSR240612 (10 mg/kg, gavage), confirming a role for B1R in this response. SSR240612 had no direct effect on body temperature in control rats (Figure6) or in STZ rats (not shown) when compared to vehicle values. The intraperitoneal injection of BK (1 mg/kg) caused a non-significant diminution (−0.6°C at five minutes) of body temperature in STZ-treated rats, excluding a possible role for B2R through a peripheral mechanism (Figure7).

### Mechanism of B1R-induced hyperthermia

**Figure 8:**
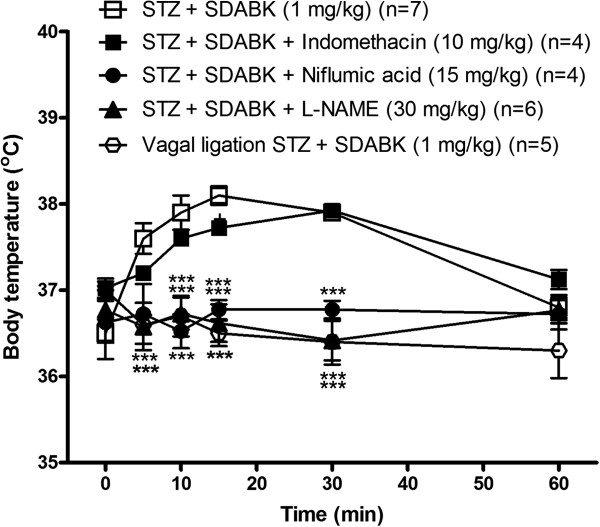
**Impact of treatments on B1R agonist-induced increased rectal temperature.** B1R agonist (SDABK, 1 mg/kg) was injected intraperitoneally in STZ rats pre-treated (2 h earlier) either with vehicle or inhibitors of COX-1 (indomethacin, 10 mg/kg, i.p.), COX-2 (niflumic acid, 15 mg/kg, i.p.) or NOS (L-NAME, 30 mg/kg, i.p.). B1R agonist-induced hyperthermia was also evaluated in STZ rats subjected to subdiaphragmatic vagal nerve ligation (14 days earlier). Statistical comparison with STZ + SDABK pretreated with vehicle (*) is indicated by *** *P* <0.001. n = 4 to 7 rats.

Selective inhibitors of NOS, COX-1 and COX-2 were pre-administered to STZ-treated rats to investigate the downstream mediators implicated in B1R-induced hyperthermia. Figure8 shows that SDABK-induced hyperthermia was prevented by a 2 h pre-treatment with either L-NAME (30 mg/kg, i.p.) or niflumic acid (15 mg/kg, i.p.) while indomethacin (10 mg/kg, i.p.) had no significant effect. Inhibitors had no direct effect on body temperature in STZ-treated rats (data not shown). Moreover, B1R-mediated hyperthermia was prevented in rats that underwent subdiaphragmatic ligation of the vagus nerve one week prior to STZ treatment (Figure8). STZ rats presented no alteration in baseline body temperature after vagal ligation. Sham-operated rats responded normally to SDABK (1 mg/kg) (data not shown).

## Discussion

In addition to providing the first evidence that B1R is expressed on peptidergic sensory C-fibers in the vagus nerve of STZ-treated rats, data uncovered a pyretic response mediated by the activation of the B1R through a vagal sensory pathway. The hyperthermic response is mediated by NO and prostaglandins (derived from COX-2). These findings are clinically relevant as kinins and B1R are associated with systemic inflammation. Thus, in addition to causing pain through activation of primary sensory fibers and microglia [8,11,13], edema and vascular hyperpermeability [17,26], and vasodilation [9,38], the kallikrein-kinin system could also contribute to hyperthermia during inflammatory processes.

### General mechanism leading to hyperthermia

Hyperthermia and fever are initiated following exposure to exogenous (bacteria, toxin) or endogenous pyrogens (pro-inflammatory cytokines (IL-1β, IL-6 and tumor necrosis factor-α (TNF-α)) [1,20,21]. The classical and controversial view of fever is that pyrogenic cytokines are mostly generated systemically and they act centrally through COX-2 dependent prostaglandin E_2_ (PGE_2_) and EP_3_ receptor in the ventromedial preoptic area (VMPO) of the anterior hypothalamus [21]. However, another theory is that the peripheral pyrogenic message is not transmitted via a humoral route but rather by the vagus nerve to the nucleus tractus solitaries, which in turn signals to the VMPO [20]. In that scenario, the contribution of PGE_2_ derived from COX-2 is essential for the activation of vagal afferents which express PGE_2_ receptors (EP_3_) while NO is released in the VMPO [20].

### Model of B1R expression

Our aim was to determine the contribution of a vagal pathway in the regulation of body temperature by B1R. Therefore, we chose an animal model known to express high level of B1R. In STZ-diabetic rats, B1R was induced by hyperglycemia-increased oxidative stress [19,26,39]. B1R was markedly expressed in various CNS and peripheral tissues [10,30,31], including primary sensory C-fibers [31]. In this model, B1R was associated with diabetic pain neuropathy [10,40], edema [41], leukocyte migration [42] vascular permeability [18,26,43,44], all cardinal signs of systemic inflammation.

### B1R-induced hyperthermia

Our data suggested that B1R-induced hyperthermia is dependent on both COX-2 and NOS activity as systemic treatment with their specific inhibitors prevented the response of the B1R agonist. Indeed, NO release has been extensively associated with B1R stimulation in STZ-diabetic rat [8,43]. NO can promote hyperthermia by activating surrounding immune cells (macrophages, neutrophils) known for their capacity to release pyrogenic cytokines (IL-1β, IL-6 and TNF-α) [45,46]. Moreover, NO can activate efferent neurons of the central nervous system, which can in turn activate either directly the preoptic area of the hypothalamus [20,46] or indirectly brain microglia and endothelial cells to generate PGE_2_[47].

The enhanced B1R expression (mRNA and protein) on sensory C-fibers of the vagus nerve and the suppression of the B1R agonist-induced hyperthermic response after ligation of the subdiaphragmatic vagus nerve strongly suggest the involvement of a vagal sensory mechanism. The nucleus of the solitary tract is known to receive sensory information from the vagus nerve and to relay it to the thermoregulatory center in the hypothalamus [20]. Additionally, B1R stimulation can release pyrogenic cytokines (IL-1β and TNF-α), NO and prostaglandins [8], that could in turn activate the vagus nerve. Thus B1R agonist could activate vagal afferents directly and indirectly through inflammatory mediators that may act synergistically to amplify the signal.

An early study reported that i.p. injected LPS induced fever and B1R mRNA expression in the rat hypothalamus. Subdiaphragmatic vagotomy blocked both fever and B1R, but not B2R gene expression, suggesting a primary role for central B1R in the early phase of fever induced by LPS [48,49]. In our study, however, the enhanced expression of B1R in the hypothalamus of STZ rats was not affected by vagal nerve ligation, providing further evidence that the pyrogenic response induced by i.p. injected B1R agonist is mediated by a peripheral B1R mechanism.

## Conclusion

This study provides the first evidence that kinin B1R can regulate body core temperature to induce fever through a vagal sensory mechanism involving prostaglandins (via COX-2) and NO. The prevention of fever may represent an additional therapeutic benefit of B1R antagonism during inflammatory processes.

## Abbreviations

BK: Bradykinin; CGRP: Calcitonin-gene-related peptide; COX-1: Cyclooxygenase-1; COX-2: COX-2, Cyclooxygenase-2; DABK: Des-Arg^9^-BK; DMSO: Dimethylsulfoxide; IL-1β: Interleukin-1β; i.c.v.: Intracerebroventricular (ly); i.p.: Intraperitoneal; B1R: Kinin B1 receptor; B2R: Kinin B2 receptor; LPS: Lipopolysaccharide; NOS: Nitric oxide synthase; NO: Nitric oxide; PBS: Phosphate buffered saline; PGs: Prostaglandins; PGE_2_: Prostaglandin E_2_; qRT-PCR: Quantitative real-time PCR; STZ: Streptozotocin; SDABK: Sar-[D-Phe^8^]des-Arg^9^-BK; VMPO: Ventromedial preoptic area.

## Competing interests

The authors declare that they have no competing interests.

## Authors’ contributions

ST, HG and RC designed the study and analyzed the data. ST, HG and JSD performed the experiments. ST drafted the manuscript. RC wrote the final version of the manuscript. All authors have read and approved the final version of the manuscript.
